# Extraction and pre-concentration of platinum and palladium from microwave-digested road dust via ion exchanging mesoporous silica microparticles prior to their quantification by quadrupole ICP-MS

**DOI:** 10.1007/s00604-015-1643-0

**Published:** 2015-09-15

**Authors:** Winfried Nischkauer, Marie-Alexandra Neouze, Frank Vanhaecke, Andreas Limbeck

**Affiliations:** Institute of Chemical Technologies and Analytics, TU Wien, Getreidemarkt 9/164-IAC, 1060 Vienna, Austria; Ecole Polytechnique, Grp Chim Solide, Lab Phys Mat Condensed, UMR CNRS 7643, 91128 Palaiseau, France; Department of Analytical Chemistry, Ghent University, Krijgslaan 281 – S12, 9000 Ghent, Belgium

**Keywords:** Dispersed particle extraction, Functionalized mesoporous silica particles, Strong anionic exchanger, Inductively coupled plasma mass spectrometry, Platinum group elements, Environmental analysis

## Abstract

We report on the use of mesoporous silica microparticles (μPs) functionalized with quarternary amino groups for the isolation of platinum and palladium tetrachloro complexes from aqueous road dust digests. The μPs have a size ranging from 450 to 850 nm and are suspended directly in the aqueous digests, upon which the anionic Pt and Pd complexes are retained on the cationic surface. Subsequently, the μPs are separated by centrifugation. Elements that cause spectral interferences in ICP-MS determination of Pt and Pd can be quantitatively removed by adding fresh 0.240 mol L^−1^ HCl to the μPs and by repeating the centrifugation step. The analyte-loaded μPs are then dissolved in 0.1 mL of 2 mol L^−1^ HF, diluted to 2 mL, and the solutions thus obtained are analyzed by quadrupole ICP-MS. This method avoids analyte elution from the sorbent. This “dispersed particle extraction” approach yielded a run-to-run relative standard deviation ≤ 5 % for Pt and ≤ 4 % for Pd (at 0.1 ng mL^−1^, *n* = 4 road dust digests). Method detection limits (expressed as concentrations in the dust samples) are 2 and 1 ng g^−1^ for Pt and Pd, respectively. The method was validated by analysis of a reference material (BCR CRM 723) and applied to the analysis of road dust samples collected in downtown Vienna. Pt and Pd concentrations in samples collected in summer and in winter were compared, with concentrations ranging from 205 to 1445 ng g^−1^ for Pt and from 201 to 1230 ng g^−1^ for Pd.

**Graphical Abstract:**
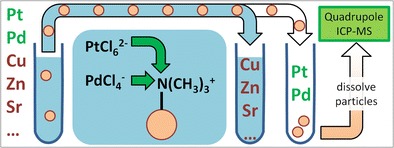
Mesoporous silica microparticles (μPs) functionalized with quarternary amino groups were used for isolating platinum and palladium from aqueous road dust digests. The μPs were suspended directly in the aqueous digests, and the analyte-loaded μPs were analyzed using “dispersed particle extraction”.

Mesoporous silica microparticles (μPs) functionalized with quarternary amino groups were used for isolating platinum and palladium from aqueous road dust digests. The μPs were suspended directly in the aqueous digests, and the analyte-loaded μPs were analyzed using “dispersed particle extraction”.

## Introduction

In order to reduce the emission of harmful by-products from the combustion of petrol in engines, automotive catalysts have been increasingly implemented during the last decades [1]. Although modern catalysts are effective in reducing CO, NO_x_ and residual hydrocarbons, they release platinum group elements (PGEs) during operation (e.g., [2, 3]). As a consequence of aerosol deposition, concentrations of platinum, palladium and rhodium in roadside soils increased over the years [4]. Studies have revealed that catalyst-borne PGEs can be taken up by plants and other species (e.g., [1, 5]). The toxicological potential of traffic-related PGEs is still not completely understood, making it necessary to study their effect on various species in exposure studies, as well as to improve analytical methods for reliable environmental monitoring of those elements.

Due to their relatively low sensitivity, inductively coupled plasma-optical emission spectrometry (ICP-OES) or atomic absorption spectrometry (AAS) are restricted to exposure studies with higher-than-natural PGE concentrations (among others [6, 7]). To conduct exposure studies with realistic concentration levels, or to analyze native environmental samples, significant analyte pre-concentration is necessary when applying ICP-OES or AAS (e.g., [8–13]).

ICP-mass spectrometry (ICP-MS) offers the required sensitivity for PGE quantification in native environmental samples, as well as multi-element capability and ruggedness [14, 15]. Nevertheless, the determination of PGEs by means of ICP-MS is a challenging task due to spectral interferences arising from ubiquitous matrix elements. In ICP-MS, the occurrence of polyatomic ions jeopardizes the accuracy of ultra-trace level PGE concentration data. The signals from ^40^Ar^65^Cu^+^ and ^40^Ar^66^Zn^+^, e.g., overlap with those from ^105^Pd^+^ and ^106^Pd^+^, respectively. The number of such polyatomic interferences, formed from elements present in the sample matrix, the solvent and/or entrained air, is long [14]. Some of these interferences cannot be avoided even when measuring at high mass resolution in sector-field ICP-MS instrumentation [16]. Given the complexity of the situation, interference-free conditions can also not readily be obtained via chemical resolution in a multipole collision/reaction cell ICP-MS [17, 18]. Considering the difficulties in overcoming spectral interferences solely by instrumental means in this context, there is a wide consensus that PGEs have to be chemically isolated prior to analysis.

Conventionally, chemical isolation of PGEs is achieved by means of co-precipitation (e.g., [19, 20]) or solid-phase extraction (SPE) [14], preceded by adequate digestion procedures [21]. Although co-precipitation is an established technique, it is cumbersome and requires costly high-purity chemicals. In contrast, SPE has the potential for higher sample throughput and requires fewer chemicals. Key principle in PGE isolation by SPE is the formation of anionic PGE chloro-complexes. Their anionic character allows separation from the predominantly cationic interferences using two approaches: i) retention of matrix cations, while PGE-containing anions pass through a strong cationic exchanger resin (SCX, e.g., [16, 22]), and ii) selective retention of PGE-containing anionic complexes, while the cationic matrix ions pass through a strong anionic exchanger resin (SAX, e.g., [20, 23, 24]), followed by the elution of the retained analytes. Due to the repeated use of SPE-columns, memory-effects are a major problem, especially in case of SAX functionalities which strongly retain PGE chloro-complexes. To minimize memory-effects, the elution is typically performed with concentrated mineral acids [23, 24] or noxious complexing agents, such as thiourea [14]. Consequently, elution of the PGEs from SAX resin is problematic in terms of safety, waste management and ICP-load.

Also in this work, strong anionic exchanger (SAX) functionalities are applied to selectively retain PGE chloro-complexes. However, to circumvent the challenges and disadvantages associated with PGE elution, the recently developed approach of “dispersed particle extraction” (DPE) [25, 26] was used. In DPE, a mesoporous sorbent-resin is suspended directly in the liquid sample. After analyte sorption, the micro-particles are separated from the surrounding liquid matrix. Due to the high surface area of the micro-particles, small amounts of the resin are sufficient for PGE retention. As a consequence, in a subsequent step, the analyte-loaded sorbent material can be dissolved directly, thus circumventing the elution step. The analytes are then introduced to an ICP-MS together with the disintegrated micro-particles.

So far, the DPE-approach achieved good results in extracting cationic metals from environmental aqueous samples [25], as well as in extracting cationic rare earth elements from saline waters [26]. Here, we have deployed the DPE-approach for the first time to the isolation of anionic platinum and palladium chloro-complexes from chemically digested road dust samples. This new approach was validated by successful analysis of reference material BCR CRM 723 (road dust) and used for analysis of road dust samples collected in downtown Vienna (Austria).

## Experimental

### Reagents and materials

In all experiments, the reagents used were of analytical grade or higher purity. The chemicals used for the synthesis of the SAX sorbent material were of synthetic grade or higher purity. Concentrated nitric acid, hydrochloric acid, hydrofluoric acid and hydrogen peroxide were purchased from Merck, Germany (www.merckmillipore.com). 1000 mg L^−1^ stock solutions of platinum, palladium, and indium in 5 % (*v/v*) HCl were obtained from Fluka, Germany (www.sigmaaldrich.com), and used for the preparation of calibration standards by dilution with 2 % (*v/v*) HCl or as internal standard. High purity water was prepared using an Easypure water system (Thermo, USA, resistivity ≥18 MΩ cm, www.thermofisher.com). BCR CRM 723 road dust reference material was obtained from IRMM (Geel, Belgium, https://ec.europa.eu/jrc/en/reference-materials).

### Instrumentation

Measurements were performed using an iCAP Qc quadrupole ICP-MS instrument (Thermo, Bremen, Germany, www.thermofisher.com), equipped with a concentric nebulizer and a quartz cyclonic spray chamber connected to the ICP-torch for sample introduction (quartz injector tube of 1.5 mm inner diameter). Sample uptake was accomplished via an ESI (Omaha, NE, USA, www.icpms.com) SC2-DX autosampler in combination with an ESI FAST sample introduction system (1 mL sample loop). Prior to each measurement session, the ICP-MS instrument settings were optimized using a solution containing 1 μg L^−1^ of indium, barium, uranium and cerium to achieve satisfying sensitivity, oxide ratios (CeO^+^/Ce^+^ < 2 %) and doubly charged ion levels (Ba^++^/Ba^+^ < 3 %). Typical operation conditions are given in Table 1. All measurements of solutions after dispersed particle extraction were performed in standard ICP-MS mode. Three isotopes of sufficient abundance were selected for each analyte. ^115^Indium was used as an internal standard. For monitoring the matrix elements, nuclides of sufficient abundance were measured in kinetic energy discrimination (KED) mode using a mixture of 7 % hydrogen in helium as collision gas at 3 mL min^−1^ and an energy barrier of –3 V.

**Table 1:** Instrumental settings of the iCAP Qc (Thermo, Bremen)

Nebulizer gas flow rate	0.95	L min^−1^
Cool gas flow rate	14	L min^−1^
Auxiliary gas flow rate	0.8	L min^−1^
Plasma power	1550	W
cone material	Nickel	
dwell time per isotope	0.01 s (4 main runs, 80 sweeps each)	
sample flow rate	0.5	mL min^−1^
Nuclides monitored	^105^Pd, ^*106*^ *Pd* ^*a*^, ^108^Pd, ^194^Pt, ^*195*^ *Pt* ^*a*^, ^196^Pt, ^115^In^b^	

^a^nuclide used for quantification

^b^internal standard

Road dust samples were digested using a microwave-assisted closed vessel treatment (Multiwave 3000, HF 100 vessels, Anton Paar, Austria, www.anton-paar.com). Separation of SAX sorbent particles from the surrounding liquid sample was done in a Heraeus Megafuge 16 centrifuge (Thermo Scientific, www.thermofisher.com), equipped with a FIBERLite F15-6x100 angular rotor (pitch angle 25°, acceleration: 17,000×*g*) using 15 mL metal-free polypropylene vials (maximum tolerable acceleration: 17,000×*g*, VWR collection, VWR, Germany, www.vwr.com).

### Synthesis and characterization of SAX micro-particles

**Fig. 1:**
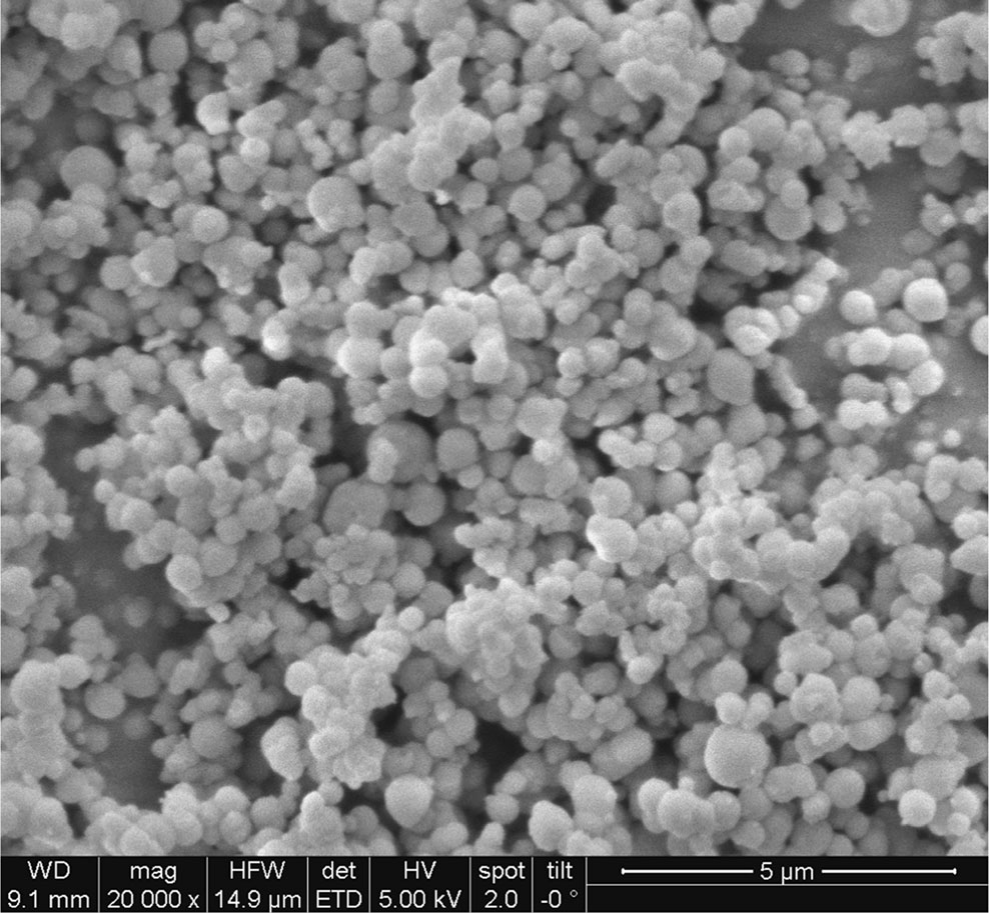
SEM micrograph of mesoporous silica particles functionalized with SAX functionalities (bar = 5 μm, acceleration voltage: 5 kV)

The ion exchanging resin with mesoporous structure was synthesized in-house according to methods described previously [25, 26] and modified with quarternary amine functionalities according to [27]. To summarize, the following synthetic steps were performed: first, sub-micron silica particles of nanometre porosity were obtained by hydrolysis of tetraethoxysilane under basic pH conditions (NH_3_) in the presence of cetyltrimethylammonium bromide (CTAB) surfactant. The micelles formed by the CTAB surfactant act as template for nano-pores. The particles were removed from the solution by centrifugation, washed with water and ethanol and calcinated at 550 °C for 5 h to remove the surfactant. In a second step, the silica surface was activated using concentrated HCl. Then, aminopropyltrimethoxysilane was added to introduce amine groups onto the surface of the particles. In a third step, the amines were quarternized with methyl iodide. The particles were characterized using nitrogen sorption at 77 K (ASAP 2000, micromeritics, USA, www.micrometrics.com) and Scanning Electron Microscopy (Quanta 200 MK2, FEI, USA, www.fei.com). The physical properties of the silica particles are: 1084 m^2^ g^−1^ specific surface area, 2.5 nm average pore diameter, and particle diameters ranging from 450 to 850 nm. The loading capacity was found to be 0.0024 milliequivalent g^−1^. This value was determined by saturating the particles with [PdCl_4_]^2−^ in 0.240 mol L^−1^ HCl and quantifying the adsorbed palladium after three washing steps. Under those conditions, analyte-concentrations of up to 100 ng mL^−1^ can therefore be extracted with constant extraction efficiency, which is far above the here investigated concentrations which are below ng mL^−1^. Saturation of the particles should therefore not be observed for the concentrations expected for environmental samples. Figure 1 shows a SEM micrograph of the mesoporous material finally obtained.

### Sample collection and digestion

Dust samples were collected in March 2011 and July 2011 in downtown Vienna, Austria (location “Museumsplatz”: 48.20370°N, 16.35923°E, 180 m above sea level). Sampling was done 1 week after the last rain or snow event from dry ground. At both sampling events, one sample was collected in a subterranean parking garage (location 1) and one sample was collected next to the street at 1 m distance from the curbstone (location 2). Sampling was achieved using a PE brush and shovel. At least 200 g of dust were collected at each location. The collected material was dried at 70 °C until constant weight, then sieved to obtain the particle fraction < 0.5 mm and thoroughly homogenized. Until further use, the samples were stored in PE plastic bags in an exsiccator over silica gel.

Solid samples (30–100 mg) were digested using a three-step microwave-assisted closed-vessel treatment, following the method suggested for BCR CRM 723 reference material in [28] with minor adaptations. A Multiwave 3000 microwave system (Anton Paar, Austria, www.anton-paar.com) in combination with high-pressure Teflon vessels was used for this purpose (heating ramp: 20 min, hold-time: 35 min, maximum power: 900 W, maximum internal temperature: 240 °C, maximum pressure: 40 bar). In the first step, a mixture of 4 mL of concentrated HNO_3_ and 2 mL of H_2_O_2_ (30 %) were added to the solid sample and the mixture thus obtained was submitted to the microwave program mentioned above. After cooling down, an additional 0.5 mL of concentrated HNO_3_ was added and the mixture was submitted to a second microwave treatment (same program as mentioned above). Finally, 3 mL of concentrated HCl and 1 mL of concentrated HF were added, and once again, the microwave digestion was carried out. This intense digestion ensured complete oxidation of the elemental carbon present in the samples and the reference material and it also allowed digestion of silicates. This microwave-program was relied on for digestion of both the reference material and the collected dust samples.

Upon completion of the microwave-assisted digestion, the clear solutions were quantitatively transferred into PTFE-beakers and the acids were evaporated at 85 °C to near-dryness. Subsequently, 5 mL of *aqua regia* were added and the samples were again evaporated to near-dryness. This procedure was repeated three times, each time with addition of 5 mL of concentrated HCl. This repeated boiling in HCl ensured conversion of Pt and Pd into their chloro-complexes [16]. Moreover, remaining hydrofluoric acid is removed by this procedure, thus avoiding possible precipitation of fluorides and destruction of silica sorbent particles in the subsequent sample pre-treatment procedure. Finally, the samples were taken up in 21 mL of 0.240 mol L^−1^ HCl and – if not analyzed immediately – stored refrigerated (4 °C) until further use. Digested samples were not stored longer than 36 h to avoid losses by sorption to plastic containers.

After each digestion run, the microwave system was cleaned by applying the same chemicals as required for one sample digestion run, whereas the PTFE-beakers were cleaned by immersion in boiling fresh *aqua regia* overnight (2 times). After cleaning, all vessels were thoroughly rinsed with high-purity water and dried under ambient conditions.

### General description of the DPE procedure

**Fig. 2:**
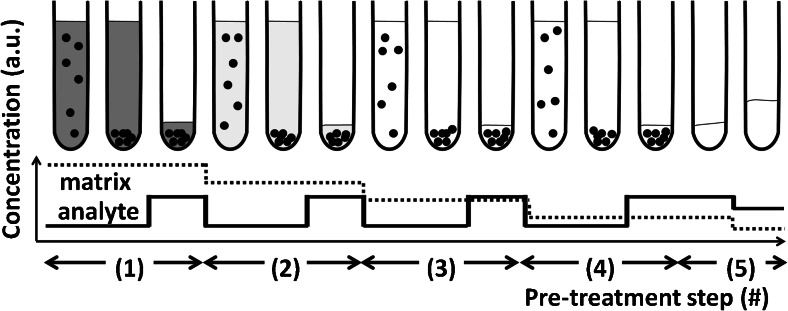
Schematic of the DPE sample pre-treatment process (black spots: SAX micro-particles). This figure is based on quantitative analyte-recoveries

Ten milliliters of sample digest (0.240 mol L^−1^ HCl) were transferred into a metal-free centrifugation tube. The optimum amount of SAX sorbent material (2 mg) was added in the form of an aqueous suspension and the sample was homogenized in an ultrasonic bath. A first centrifugation step was performed at 17,000×*g* for 10 min. The particles were found to be strongly compressed onto the walls and the bottom of the centrifuge tube and therefore, it was possible to swiftly decant the supernatant solution (see Fig. 2(1)). For further removal of matrix constituents, the precipitate was re-suspended in 10 mL of 0.240 mol L^−1^ HCl. After manual shaking and ultrasonic agitation, the centrifugation step was repeated and the supernatant solution discarded (Fig. 2(2)). Thereby, matrix components that remained on the particles during the first decantation-step can be removed. This washing cycle was repeated two more times (Fig. 2(3) and (4)). Finally, the mesoporous particles were destroyed by adding 0.1 mL of a mixture containing 2 mol L^−1^ HF and 1.2 mol L^−1^ HCl. After adding indium as internal standard, the solutions were diluted to 2 mL using high purity water (Fig. 2(5)). As schematically depicted in Fig. 2, this procedure yields a stepwise removal/dilution of any matrix constituents that do not bind to the SAX-sorbent material. Contrarily, analytes that are adsorbed onto the sorbent resin remain at constant concentration and finally become pre-concentrated. The theoretical enrichment factor depends on the ratio of starting volume to final volume and was 5.

## Results and discussion

### Optimization of dispersed particle extraction

During initial experiments, it was found that the recovery of rhodium was very low, compared to Pt and Pd. When performing a reduction with SnCl_2_, recoveries for Pt, Pd, and Rh improved. However, it was found that the high concentrations of Sn that were added to the samples resulted in major spectral interferences. On the one hand, the internal standard was influenced (^115^In^+^ is affected by the isobaric interference of ^115^Sn^+^). This problem may be solved by using an other element for internal standard. Yet, on the other hand, the solutions treated with Sn resulted in a significantly enhanced Pt-blank. This may be caused by the formation of SnArCl^+^ in the presence of hydrochloric acid medium resulting in polyatomic ions of masses 194, 195, 196. Therefore, as the addition of Sn produced severe problems, it was opted for not analyzing Rh and for optimizing the method for Pt and Pd only, using hydrochloric acid medium.

Three factors affect the retention of Pt and Pd on SAX micro-particles: (i) the pH, (ii) the amount of micro-particles, and (iii) the time allowed for interaction of the analytes with the sorbent material (“interaction time”). To a certain extent, these factors are related to one another, and therefore, an iterative optimization process was done. First, the optimum pH value was found by providing 10 mL of a solution containing 0.1 ng (Pt, Pd) mL^−1^ with varying concentrations of HCl (0.012 to 0.360 mol L^−1^) and using a fixed amount of 2 mg sorbent material. An optimum was found at 0.240 mol L^−1^ of hydrochloric acid. Then, the amount of sorbent material was varied between 0.5 and 5 mg using the same experimental set-up as mentioned above, and using 0.240 mol L^−1^ HCl medium. At 2 mg of SAX sorbent material, a plateau was reached, i.e., no further improvement of the recovery was observed at higher amounts of sorbent material, and therefore, 2 mg were used in the further experiments. The interaction time in the ultrasonic bath (time between the addition of sorbent and the start of the first centrifugation step) was found to have no observable effect on the recovery (times of 1, 2, 5, 10, and 15 min were investigated), however, without ultrasonication, slightly lower recoveries were observed. Therefore, 5 min of ultrasonication were used in all further experiments. With the optimum interaction time and particle amount, a final investigation of the influence of sample acidity on recovery was carried out to ascertain that optimum conditions were indeed used (optimum: 0.240 mol L^−1^ HCl).

### Evaluation of spectral interferences

**Fig. 3:**
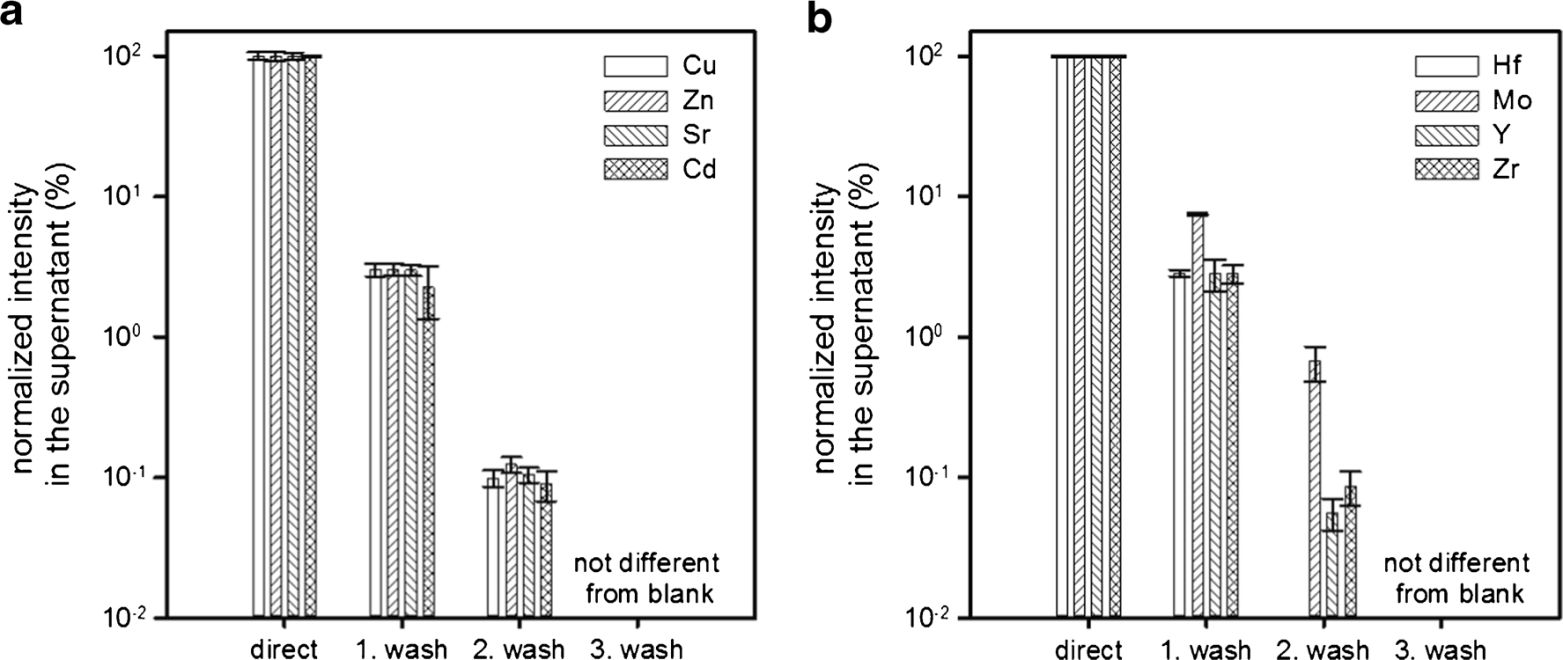
Three successive washing steps result in an effective removal of potentially interfering elements (concentrations at the beginning: 10,000 ng mL^−1^ for Cu, Zn, Sr, 1000 ng mL^−1^ for Y, Zr, and 100 ng mL^−1^ for Cd, Hf, Mo, *error bars* represent the standard deviation of *n* = 3 replicates)

As discussed in the introduction, the removal of spectral interferences is a very important prerequisite for obtaining correct quantitative results. In the road dust CRM (BCR 723), many parent nuclides can be found which potentially cause such spectral interferences (see [14] for an extensive list). Therefore, the effectiveness of removing Cu, Zn, Sr, Cd, Hf, Mo, Y, and Zr from the samples when applying the proposed DPE-method was investigated in more detail. To this end, solutions containing those elements in concentrations expected after digesting BCR 723 road dust were prepared from single-element standards (concentrations ranging from 10,000 to 100 ng mL^−1^, depending on the element). To simulate the digestion, the solutions were mixed with aqua regia and HF and evaporated repeatedly to near-dryness as described above (see section “sample collection and digestion”). Finally, the samples were taken up in 0.240 mol L^−1^ HCl and the proposed DPE sample pre-treatment process was carried out three times. The supernatants resulting from each of the three pre-treatment steps were analyzed for their respective element concentrations. As can be seen in Fig. 3, the concentrations of all elements decrease with every washing step, namely to 2.3–7.5 % of the initial concentration after the first washing step (signals obtained from the first supernatant correspond to the digest without any pre-treatment and are normalized to 100 %), to 0.01–0.2 % after the second washing step, and to levels not significantly different from the blank after the third washing step. Therefore, three washing steps were used in all further experiments.

### Analyte recovery and figures of merit

Typical recoveries for aqueous standard solutions, as well as for road dust digests and different dilutions thereof were in the range of 64–80 % for Pd and 21–35 % for Pt. These values represent the variability of the method under different matrix conditions (aqueous standards, digested samples) and different analyte-concentrations, using a three-step sample pre-treatment scheme. It should be noted that the goal of the approach presented here was rather to remove interfering matrix elements than to obtain quantitative recoveries for Pt and Pd.

From the analyte-recovery and the theoretical pre-concentration factor of 5 (10 mL starting volume, 2 mL final volume), the pre-concentration factors for Pd and Pt were determined to range between 3.2–4 and 1.05–1.75, respectively.

For quantification purposes, four-point standard addition was used for every sample. Adequate amounts of Pt and Pd were spiked to the digests prior to performing the DPE pre-treatment.

The process of digestion, analyte sorption, and step-wise matrix removal typically yielded a reproducibility of ≤ 5 % relative standard deviation (RSD) for Pt and ≤ 4 % RSD for Pd (at 0.1 ng mL^−1^, *n* = 4 road dust digests). Internal standardization with indium was carried out to compensate for potential instrument instability and/or signal drift. Detection limits were comparable to typical values obtained with quadrupole ICP-MS instrumentation (2 pg mL^−1^ for Pt and 1 pg mL^−1^ for Pd, calculated from 8 blank solutions pre-treated independently with the proposed DPE-procedure, 3 s-criterion). The method quantification limits in the native dust samples were 2 and 1 ng g^−1^ for Pt and Pd, respectively.

The step-wise removal of interfering elements, as shown in Fig. 3, allowed for a successful quantification of palladium and platinum in BCR CRM 723. Found concentrations were 80.2 ± 0.7 ng g^−1^ for Pt and 6.2 ± 2.6 ng g^−1^ for Pd (*n* = 4, results given as average and standard deviation of 4 sample digests). These values are in good agreement with the certified values of 81.3 ± 2.5 ng g^−1^ Pt and 6.1 ± 1.9 ng g^−1^ Pd.

#### Analysis of road dust samples

**Fig. 4:**
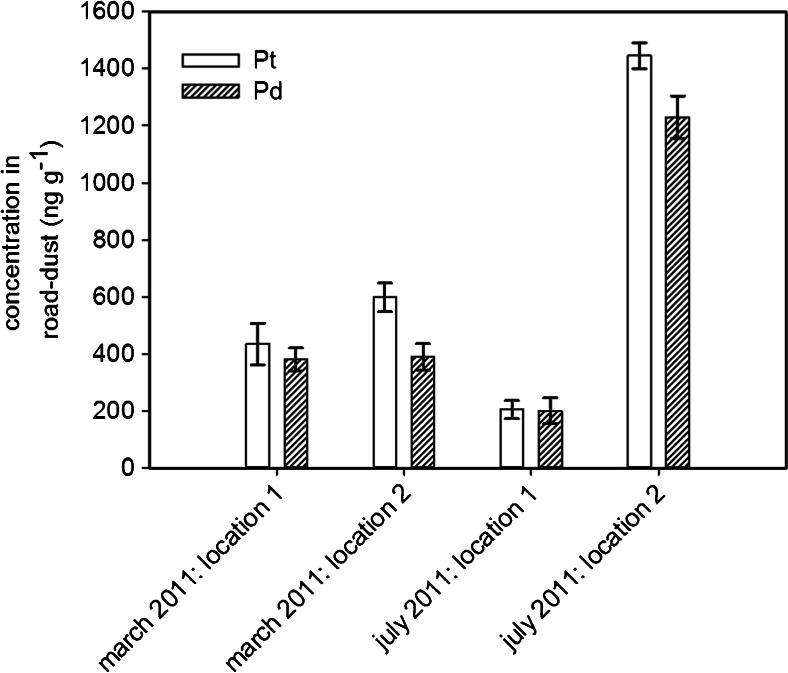
Found concentrations in roadside-dust (location 1: subterranean parking garage, location 2: road-side, all concentrations above method quantification limit, *error bars* represent the standard deviation of *n* = 2 replicates)

The dust samples collected in March and July 2011 were digested, the optimized DPE-procedure was applied, and Pt and Pd were quantified by means of quadrupole ICP-MS in standard mode. The results are summarized in Fig. 4. All concentrations were above the method quantification limit. Found concentrations for platinum ranged from 205 to 1445 ng g^−1^, which is in the same order of magnitude as reported in the literature [29–32]. Concentrations of palladium ranged from 201 to 1230 ng g^−1^, which is also in agreement with reported data [31, 32].

The roadside concentrations (location 2) in July were found to be higher than those observed in March. Dust sources, such as gravel used for winter service and dust caused by abrasion of the road-surface, are more prominent during winter and result in a higher overall dust load. Provided that the amount of PGEs emitted by the traffic is constant over the year, the higher dust load in winter could therefore result in a dilution of Pt and Pd.

The elemental Pt/Pd ratio was found to be 1.2 ± 0.2 (average and standard deviation for the four dust samples). Kanitsar et al. [33] have found a Pt/Pd ratio of 2.7 ± 0.6 in total suspended matter (airborne dust), whereas Petrucci et al. [34] have found Pt/Pd ratios ranging from 0.1 to 2.4 in PM_10_ airborne particulate matter. Earlier investigations, which are summarized in [29], reported on much higher Pt/Pd ratios of up to 37.5. Changes in the composition of the catalyst material (also reflecting changes in raw material prices) also contribute to this trend.

## Conclusions

We have investigated a novel isolation procedure for platinum and palladium chlorocomplexes from aqueous road dust sample digests. The method of dispersed particle extraction was successfully adapted to allow for the retention of anionic Pt and Pd chlorocomplexes. Method quantification limits of 2 and 1 ng g^−1^ for Pt and Pd, respectively, are comparable to those of existing procedures [14, 15, 35]. By fully exploiting the potential of dispersed particle extraction in terms of pre-concentration, higher pre-concentration factors would be feasible.

The advantages over conventional solid-phase extraction are that (i) no conditioning of the sorbent material is necessary, that (ii) the often troublesome elution step is avoided, and that (iii) memory effects are fully circumvented by using new sorbent material for every experiment. Moreover, the approach can be adjusted to the analytical problem at hand to increase the enrichment factor or to improve the separation efficiency, either by adjusting the start and end volumes, or by changing the number of washing steps. The amount of sorbent material required for one analysis (2 mg) rationalizes the in-house synthesis of the material, and certainly undercuts the costs for single-use solid-phase extraction columns. The method presented here facilitates sample pre-treatment and improves sample throughput. Importantly, no significant blank issues were observed and the lifetime of cones and nebulizer were not affected, even though rather high amounts of silicon in the form of dissolved silica particles were introduced into the ICP-MS instrument over longer time.  If the presence of Si forms a problem in future applications, one may use organic mesoporous particles. These will not cause a background of mineral elements.
